# Inflammatory markers in autoimmunity induced by checkpoint inhibitors

**DOI:** 10.1007/s00432-021-03550-5

**Published:** 2021-04-10

**Authors:** Beate Husain, Michael Constantin Kirchberger, Michael Erdmann, Sabine Schüpferling, Amir-Reza Abolhassani, Waltraud Fröhlich, Carola Berking, Lucie Heinzerling

**Affiliations:** 1 Department of Dermatology, Universitätsklinikum Erlangen, Ulmenweg 18, 91054 Erlangen, Germany; 2 Friedrich-Alexander University Erlangen-Nuremberg, 91054 Erlangen, Germany; 3 Department of Cardiology, Klinikum Forchheim, 91301 Forchheim, Germany; 4 Comprehensive Cancer Center Erlangen-European Metropolitan Area of Nuremberg (CCC ER-EMN), 91054 Erlangen, Germany; 5 Deutsches Zentrum Für Immuntherapie (DZI), Friedrich-Alexander University Erlangen-Nuremberg and Universitätsklinikum Erlangen, 91054 Erlangen, Germany

**Keywords:** Immune-related adverse events, Cytokines, Immune checkpoint inhibitors, Melanoma

## Abstract

**Purpose:**

Immune checkpoint inhibitors (ICI) are highly effective in several cancer entities, but also invoke a variety of immune-related adverse events (irAE). These are mostly reversible, but can be life-threatening or even fatal. Currently, the pathogenesis is not fully understood, but crucial for effective treatment. Prediction and early detection of irAE could be facilitated and treatment optimized if relevant biomarkers and effector mechanisms were better characterized.

**Methods:**

This study included a total of 45 irAE in patients with metastatic melanoma who were treated with ICI. All patients underwent a complete work-up with exclusion of other causes. Longitudinal blood samples were analyzed for a panel of soluble markers and compared to baseline and to patients who did not experience any irAE. Measurements included LDH, interleukin (IL)-6, IL-1β, IL-17, C-reactive protein (CRP) and tumor necrosis factor (TNF)-alpha as well as tumor markers S100 and melanoma inhibitory activity (MIA).

**Results:**

During the early onset of irAE increases in serum IL-6 (from mean 24.4 pg/ml at baseline to 51.0 pg/ml; *p* = 0.003) and CRP (from mean 7.0 mg/l at baseline to 17.7 mg/l; *p* = 0.001) and a decrease in MIA (from mean 5.4 pg/ml at baseline to 4.8 pg/ml; *p* = 0.035) were detected. No changes in IL-17 were noted. These effects were observed for irAE of different organ systems.

**Conclusion:**

Increases of a combination of IL-6 and CRP serum levels can be used for the early detection of irAE and tailored management. Interestingly, changes in MIA serum levels also correlate with irAE onset.

## Introduction

Immune checkpoint inhibitors (ICI) have changed the clinical landscape of cancer therapy (Ugurel et al. 2016), which started with ipilimumab, a monoclonal antibody targeting cytotoxic T-lymphocyte antigen 4 (CTLA-4). It was the first ICI therapy approved by the FDA for metastatic melanoma in 2011. This was followed by antibodies targeting PD-1 (pembrolizumab, nivolumab, cemiplimab), PD-L1 (atezolizumab, avelumab, durvalumab) and again CTLA-4 (tremelimumab) that have shown efficacy in various tumor entities. The improvement of survival in patients treated with ICI, however, is counterbalanced by the induction of a plethora of immune-related adverse events (irAE) that can be fatal (Heinzerling and Goldinger 2017; Sosa et al. 2018; Wang et al. 2018). Early clinical trials already documented irAE that could manifest in different organ systems (Beck et al. 2006). Typically, the gastrointestinal system (e.g., colitis), skin (e.g., pruritus, exanthema), the endocrine system [e.g., thyroiditis, hypophysitis (Chae et al. 2017)], liver (e.g., hepatitis), lung (e.g., pneumonitis), and the musculoskeletal system [e.g., myositis, myopathy (Cappelli et al. 2017; Haikal et al. 2018; Moreira et al. 2019; Voskens et al. 2013)] are affected. Less frequently, neurological [e.g., encephalitis, polyneuropathy; (de Maleissye et al. 2016)] or cardiologic side effects (Heinzerling et al. 2016) can be induced. Especially affection of the nervous and cardiovascular system is associated with high morbidity and mortality (Wang et al. 2018).

With the growing use of ICI in more and more tumor entities, patient subgroups and earlier tumor stages, as well as in patients with various co-morbidities, the timely recognition of irAE and adequate management are increasingly important. Furthermore, a better understanding of the underlying pathomechanisms will improve the physicians’ capacity to efficiently manage irAE. Several mechanisms of ICI-induced autoimmunity have been described to be triggered by the inhibition of negative costimulatory signals (Urwyler et al. 2020). Patients with irAE develop an early enhanced diversification (Oh et al. 2017) and activation of the peripheral T-cell pool (Robert et al. 2014) and a broadening in reactive CD8^+^ T-cell response, in which the newly detected T lymphocytes had a higher tumor recognition potential (Kvistborg et al. 2014). This corresponds to a recent meta-analysis which has shown that the occurrence of irAE during an ICI treatment correlated with a better clinical outcome (Haratani et al. 2020). Patients with malignancies with a high tumor mutational burden, such as melanoma or non-small cell lung cancer, who had been treated with anti-PD-1 therapy could run a higher risk of developing irAEs due to a higher number of presented neoantigens and, therefore, increased risk that T cells cross-react with wildtype instead of neoantigens (Bomze et al. 2019). Furthermore, autoantibodies and direct binding in normal tissues has been described (Postow et al. 2018). At the same time, significant early changes in the B-cell pool of ICI patients with irAE have been observed including a drop in the total number of circulating B cells, while CD21^low^ B cells and plasmablasts surged during combined ICI regimes (Das et al. 2018), as well as the induction of autoantibodies (here predominantly against thyroid tissue) (de Moel et al. 2019). Another mechanism of irAE includes the direct binding of ICI to normal human tissues, for example of CTLA-4 in the pituitary gland. The administration of ipilimumab led to the production of pituitary-specific antibodies, complement activation and, therefore, hypophysitis (Iwama et al. 2014) and potentially anti-PD1, anti-PD-L1 expressed in muscle tissue that could trigger irMyositis (Touat et al. 2017).

Investigating the pathways of irAE cannot only help for early detection but also for tailored treatment. Thus, we specifically investigated cytokines for which antibodies are approved for application in patients which would enable rapid translation into clinical practice like interleukin-1 (IL-1), interleukin-6 (IL-6), interleukin-17 (IL-17), and tumor necrosis factor alpha (TNF-α). Our study investigated whether levels of IL-1β, IL-6, IL-17, and TNF-α were elevated during episodes of irAE in patients with metastatic melanoma undergoing checkpoint inhibitor therapy, and, therefore. could be used for monitoring of irAE during ICI therapy as well as targets for treating irAE.

## Materials and methods

### Patients and episodes

Patients were recruited from the outpatient clinic at the skin cancer center of the University Hospital Erlangen, Germany. Inclusion criteria comprised a diagnosis of metastatic cutaneous or uveal melanoma, treatment with an immune checkpoint inhibitor (ICI) and at least one episode of irAE. Patients with pre-existing autoimmune diseases and preceding or concomitant infections were excluded. To ensure reliable irAE detection, all patients were screened for and asked to report any symptoms prior and during ICI therapy. When symptoms evolved a thorough work-up to exclude other causes was performed to safely rule out infection, progression of disease or secondary pathologies.

In total, 80 samples from 16 patients, who were treated with ICI therapy, were examined. From those 16 patients, 13 patients (12 with cutaneous melanoma, 1 with uveal melanoma) experienced irAE with a total of 45 episodes of irAE (68 serum samples) and were compared to 3 control patients (12 serum samples) without any observed irAE while undergoing one or more ICI treatment regimes. ICI regimes included ipilimumab (*n* = 4), pembrolizumab (*n* = 8), nivolumab (*n* = 1), and combination therapy with ipilimumab and anti-PD1 antibody (*n* = 10).

The study was approved by the local ethics committee of the Friedrich-Alexander University Erlangen-Nuremberg.

### Serum samples and measurements

Serum samples were taken between 04/2014 and 07/2017 for routine safety analysis before, at the start and during ICI treatment. All samples were stored at − 80 °C. Tumor markers S100 and MIA, as well as CRP and LDH were assessed for routine analysis. IL-1β (RayBio, Norcross, GA, USA), IL-6 (RayBio), IL-17 (RayBio) and TNF-α (Cusabio, Wuhan, China) were measured by ELISA according to manufacturer’s instructions. Samples were taken at four time points, namely at or shortly before the beginning of ICI therapy (baseline), 2 weeks before (pre-irAE), during (acute irAE) and 2 weeks after (post-irAE) the acute clinical presentation of irAE symptoms.

### Data analyses

Data analysis and statistical tests were performed using IBM SPSS Statistics, Version 26.0.0.0. Besides descriptive statistics Shapiro–Wilk test and Wilcoxon signed-rank test were used to analyze the collected data. *p* < 0.05 was considered to be statistically significant (*) and *p* < 0.01 highly significant (**). Graphs were generated via SPSS Statistics (Version 26) and Microsoft Excel 2013.

## Results

To gain insights into the pathomechanisms as well as potential treatment options of immune-mediated adverse events induced by ICI longitudinal serum samples of a total of 45 irAE were analyzed for soluble markers and compared to baseline as well as to patients treated with ICI who did not experience any irAE. Adverse events encompassed the gastrointestinal system with colitis, hematochezia, sigmadiverticulitis, pancreatitis and lipase/amylase increases and type C gastritis (25%), fatigue (11%), exanthema (11%), musculoskeletal symptoms (9%), hepatitis (7%), neuropathic pain (7%), fever (4%), lacrimal gland prolapse (2%), vitiligo (2%) and nephritis (2%). Endocrine adverse events, including hypophysitis (9%), thyroiditis (9%), and diabetes mellitus type I (2%) made up 20% of the documented adverse events. The median time between the start of the first ICI therapy and the first manifestation of an irAE was 42 days (range 21–217 days).

**Fig. 1:**
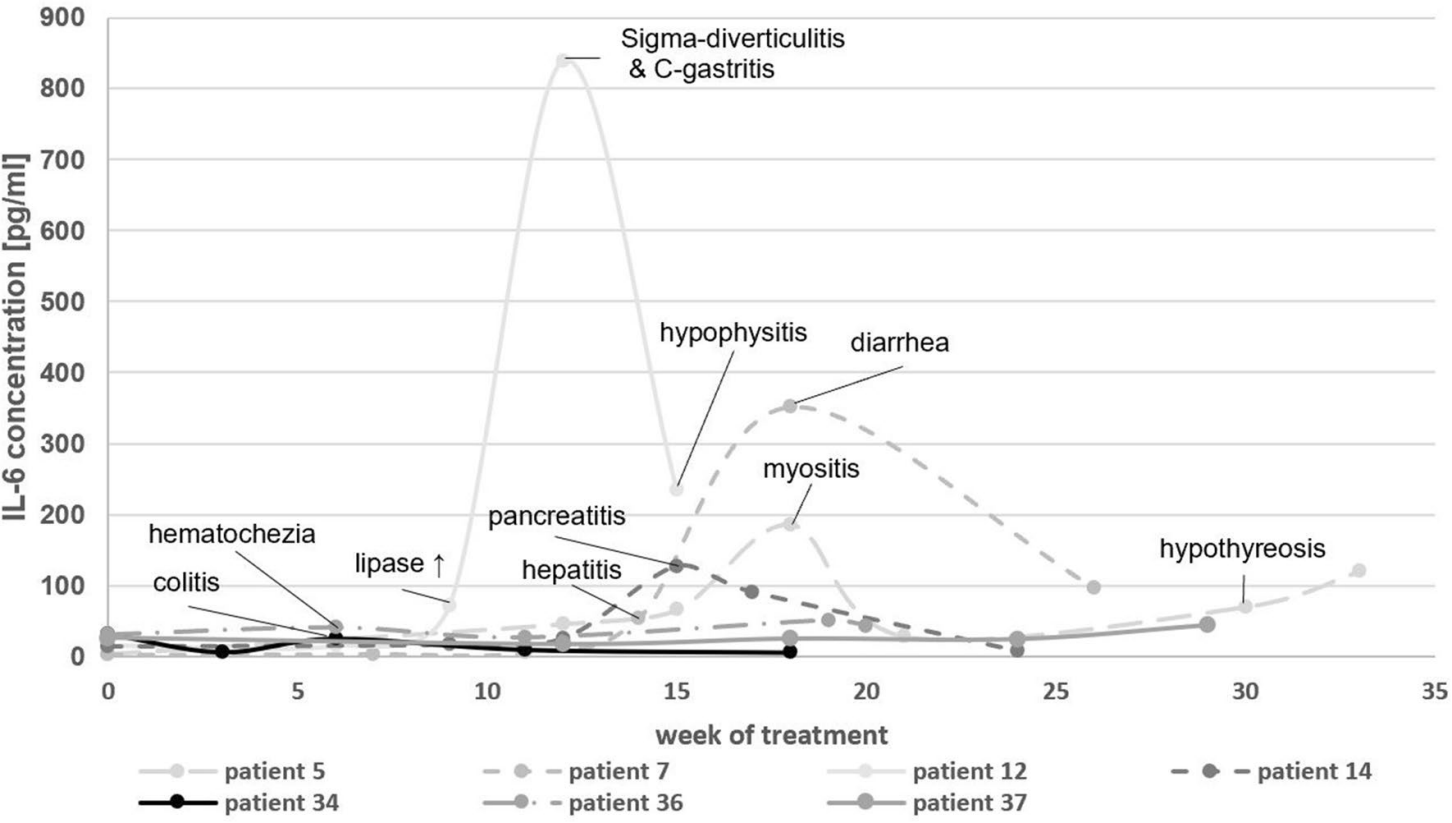
Longitudinal IL-6 concentrations in the course of immune checkpoint inhibitor therapy. The occurrence of an acute irAE is highlighted by the observed symptoms during those time periods

**Fig. 2:**
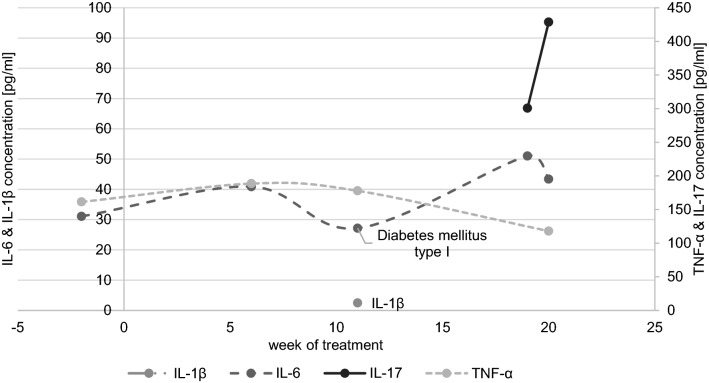
IL-1β, IL-6, IL-17, and TNF-α concentrations in the course of immune checkpoint inhibitor therapy for patient 36 with metastatic uveal melanoma. The occurrence of an acute irAE (diabetes mellitus type I) is annotated

Elevations in IL-6 serum concentrations were detected in 15 out of 21 episodes during the acute clinical manifestation of a variety of symptoms, which were consistent with irAEs. The development of IL-6 concentrations of seven exemplary patients according to their occurring irAE is depicted in Fig. 1. IL-1 was also included to determine its role as a potential mediator of irAE. All samples measured for IL-1β, except for one sample (patient 36) were below the limit of detection (< 1.02 pg/ml). Similarly, IL-17 concentrations were measured in 8 patients, totaling 40 samples. All samples, except for two samples (both patient 36) were below the limit of detection (< 93.75 pg/ml). Patient 36 was treated with a combination of ipilimumab and pembrolizumab for uveal melanoma and developed irAEs 9 weeks after the beginning of ICI therapy, specifically hematochezia and diabetes mellitus type I in week 11 (after week 11 both checkpoint inhibitors were paused). During the acute irAE phase an increased IL-1β concentration (week 11: 2.47 pg/ml) was measured, whereas IL-6 concentrations were relatively low and did not rise during the acute irAE. During the post-irAE phase increased levels of IL-17 (week 19: 300.7 pg/ml; week 20: 428.6 pg/ml) were detected. Measurements of TNF-α showed a slight increase in the pre-irAE (baseline: 161.4 pg/ml; week 6/pre-irAE: 188.5 pg/ml) and a mild decrease during the acute irAE phase (week 11/acute irAE: 177.9 pg/ml) with a decrease afterwards (week 20/post-irAE: 118.0 pg/ml). The measured cytokine concentrations for patient 36 are shown in Fig. 2.

**Fig. 3:**
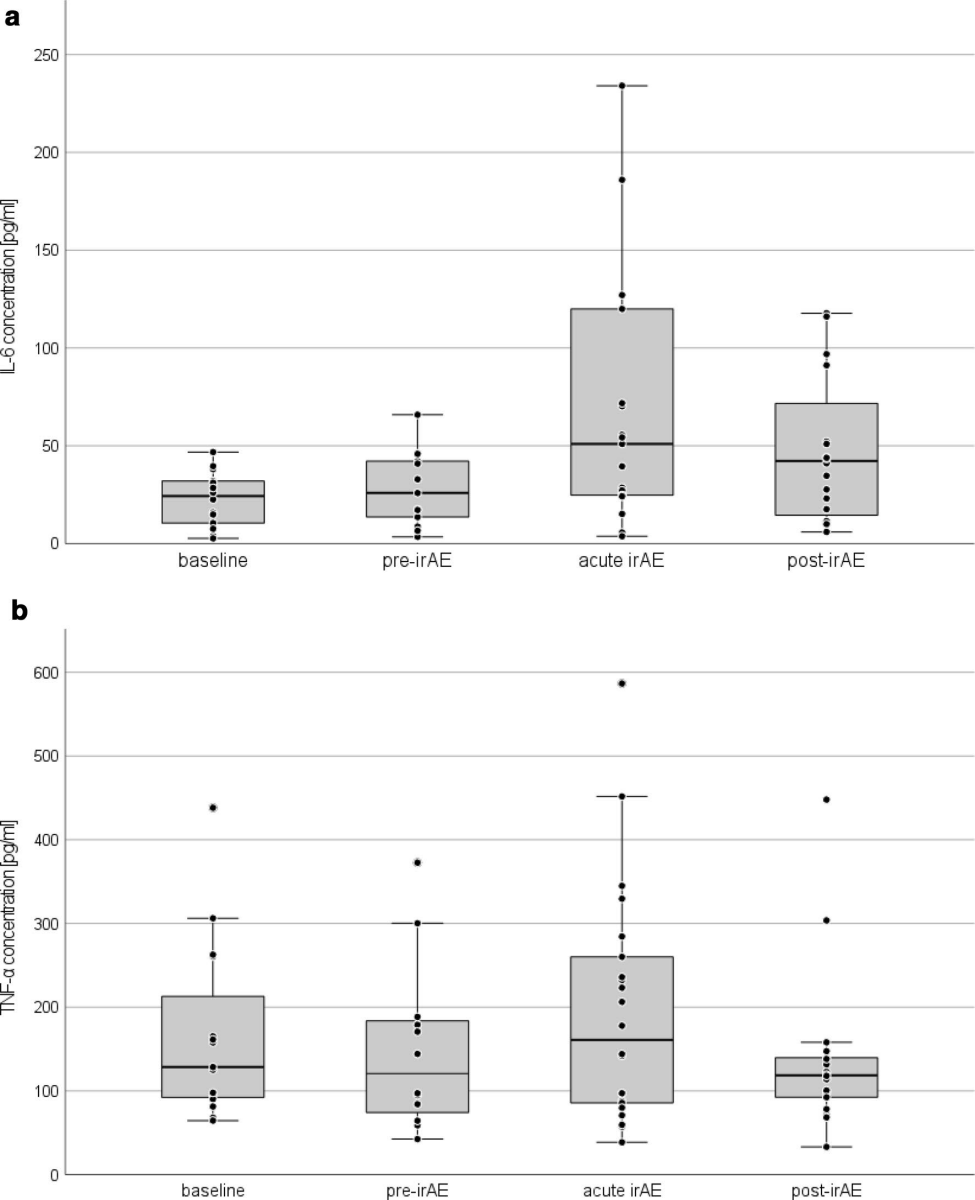
**a** IL-6 serum concentrations before, during and after irAE, with a highly significant increase during acute irAE (*p* = 0.003). Three data points were not included in this diagram (acute irAE: 351.4 pg/ml and 838.6 pg/ml; post-irAE: 286.1* pg/ml) for better graphic presentation. **b** TNF-α serum concentration before, during and after irAE, with significantly lower levels of TNF-α during the post-irAE phase (*p* = 0.022)

For each measured parameter median, range and number of samples are displayed in Table 1, as well as in boxplots for IL-6 (Fig. 3a) and TNF-α (Fig. 3b). To assess whether markers significantly differed before and during irAE, a Wilcoxon signed-rank test was performed (Table 2). During the pre-irAE phase, the only biomarker, that was significantly increased, was LDH (*p* = 0.013) while during the acute irAE phase IL-6, as well as CRP increased highly significantly (IL-6: *p* = 0.003; CRP: *p* = 0.001) and MIA values decreased significantly (*p* = 0.035). In the post-irAE phase we could measure significant decreases in TNF-α (*p* = 0.022), S100 (*p* = 0.037) and MIA (*p* = 0.021) compared to levels at baseline. In control patients no significant differences of IL-6 and TNF-α during course of ICI therapy were observed.

**Table 1:** Serum measurements and *p* values from Wilcoxon signed-rank test over time courses of irAE

	Baseline		Pre-irAE		Acute irAE		Post-irAE	
	Median (range)	*n*	Median (range) *p* value	*n*	Median (range) *p* value	*n*	Median (range) *p* value	*n*
IL-6 [pg/ml]	24.4 (2.7–46.8)	14	25.9 (3.5–65.9) 0.422	13	51.0 (3.8–838.6) 0.003**	21	42.2 (6.0–286.1) 0.073	20
TNF-α [pg/ml]	128.6 (64.6–438.3)	15	120.8 (42.5–372.7) 0.388	12	160.1 (38.7–586.7) 0.306	22	118.7 (33.2–448.0) 0.022*	18
S100 [µg/l]	0.07 (0.04–0.25)	15	0.08 (0.03–1.16) 0.328	31	0.07 (0.03–1.53) 0.170	39	0.06 (0.03–4.35) 0.037*	31
MIA [pg/ml]	5.4 (1.7–24.4)	15	6.1 (0.5–21.9) 0.128	31	4.8 (1.0–29.0) 0.035*	38	3.9 (0.9–34.2) 0.021*	29
CRP [mg/l]	7.0 (1.4–26.3)	12	5.7 (0.8–119.9) 0.575	13	17.7 (0.2–217.5) 0.001**	39	9.1 (0.3–87.7) 0.859	29
LDH [U/l]	301 (162–508)	16	309 (174–740) 0.013*	29	282 (197–670) 0.116	39	282 (161–607) 0.899	31

**Table 2:** Results of the performed Wilcoxon signed-rank test for paired samples (baseline vs. measured variable)

	Pre-irAE	Acute irAE	Post-irAE	Control
IL-6	0.422	0.003**	0.073	0.066
TNF-α	0.388	0.306	0.022*	0.953
S100	0.328	0.170	0.037*	
MIA	0.128	0.035*	0.021*	
CRP	0.575	0.001**	0.859	
LDH	0.013*	0.116	0.899	

Significant values (*p* ≤ 0.05) are highlighted as *, whereas highly significant (*p* ≤ 0.01) values are highlighted as **

Similarly, Wilcoxon signed-rank tests were performed for three specific irAE types (gastrointestinal, endocrine, musculoskeletal; Table 3). Significant increases could be seen during the acute phase in for CRP (endocrine: *p* = 0.004) and IL-6 serum concentrations (gastrointestinal: *p* = 0.037/ endocrine: *p* = 0.036/ musculoskeletal: *p* = 0.028). MIA exhibited diverse results with significant decreases during the pre-irAE (musculoskeletal: *p* = 0.012), acute (musculoskeletal: *p* = 0.003) and post-irAE phase (endocrine: *p* = 0.012). In gastrointestinal samples significant changes could be shown for LDH (pre-irAE: *p* = 0.025) and S100 (post-irAE: *p* = 0.022). No significant increases could be shown for TNF-α.

**Table 3:** Results of the performed Wilcoxon signed-rank test for paired samples (baseline vs. measured variable) for the three most common types of irAE (gastrointestinal, endocrine, musculoskeletal), which occurred in our study cohort

	Gastrointestinal irAE			Endocrine irAE			Musculosceletal irAE		
	Pre-irAE	Acute irAE	Post-irAE	Pre-irAE	Acute irAE	Post-irAE	Pre-irAE	Acute irAE	Post-irAE
IL-6	0.575	0.037*	0.214	0.123	0.036*	0.037*	0.109	0.028*	Not sufficient data
TNF-α	0.735	0.674	0.086	0.484	0.314	0.139	0.180	1.000	
S100	0.776	0.789	0.022*	1.000	0.166	0.087	0.611	0.196	
MIA	0.643	0.722	0.660	0.306	0.149	0.012*	0.018*	0.003**	
CRP	0.892	0.004**	0.306	0.686	0.131	0.975	0.465	0.060	
LDH	0.025*	0.875	0.408	0.084	0.132	0.798	0.161	0.130	

Significant differences (*p* ≤ 0.05) are highlighted as *, whereas highly significant (*p* ≤ 0.01) differences are highlighted as **

## Discussion

In this study we investigated inflammatory markers before, during and after occurrence of irAE to better understand the pathogenesis of ICI-induced irAEs, which is crucial for monitoring and managing these patients adequately. We demonstrate a distinct pattern of changes dependent on the phase of an irAE with an increase of IL-6 and CRP as well as a decrease in MIA at the time of the clinical manifestation of an acute irAE, whereas LDH was increased in the pre-irAE phase. In the post-irAE phase decreases of S100 and MIA were detected whereas for IL-17, a cytokine that is commonly elevated in inflammatory bowel diseases, no significant differences were detected in our cohort. Interestingly, another study of 35 patients treated with ipilimumab for melanoma in a neoadjuvant setting found that baseline levels of IL-17 were associated with the occurrence of irAE in 20 patients (Tarhini et al. 2015).

The role of IL-6 and irAE is still controversial. While some studies showed that high IL-6 was associated with low toxicity (Chaput et al. 2017; Valpione et al. 2018), others found that an increase of IL-6 was associated with an increase in irAE (Tanaka et al. 2017; Phillips et al. 2019) although only for cutaneous irAE. We extend these findings to irAE of different organ systems. Within our cohort IL-6 was significantly increased at the early onset of irAEs, but no correlations with IL-1β, IL-17, and TNF-α were seen. A separate analysis of three irAE types (gastrointestinal, endocrine, musculoskeletal), revealed significant increases in IL-6 serum concentrations independent of the organ system involved. Lim et al. (2019) found 11 cytokines (G-CSF, GM-CSF, Fractalkine, FGF-2, IFN-α2, IL12p70, IL-1α, IL-1β, IL1-RA, IL-2, and IL-13) which could be used for the prediction of an irAE, and developed the CYTOX score for a more objective evaluation of irAE in patients treated with a combination of anti-CTLA-4 and anti-PD-1 antibodies. Our study adds IL-6 and CRP as relevant biomarkers for irAE. We, therefore, suggest an irAE fingerprint, which should include CRP and IL-6 serum levels, since these parameters are readily available and correlate with irAEs.

First-line therapy of irAEs comprises the application of corticosteroids (Haanen et al. 2017; Heinzerling et al. 2019; Puzanov et al. 2017), which also induce a decrease in IL-6 and CRP levels (Yoshino et al. 2019). For steroid-refractory cases, the administration of anti-TNF-α antibodies (e.g., infliximab) is recommended (Badran et al. 2019). Anti-TNF-α antibodies induce a decrease of IL-1β, IL-6, IL-8 (Nesbitt et al. 2007; Ringheanu et al. 2004), IFN-γ, IL-13, IL-17A, TNF-α (Dahlén et al. 2013), and GM-CSF (Agnholt et al. 2004) and suppress CD4^+^ and CD8^+^ T-cell proliferation (Dahlén et al. 2013). However, there are also cases that do not respond to second-line immunosuppression (Lankes et al. 2016). For these, various treatment attempts exist including mycophenolate mofetil, cyclosporine A, or azathioprine (Heinzerling et al. 2019). Since our data clearly show an increase of IL-6, anti-IL-6-receptor (anti-IL-6R) antibodies like tocilizumab or sarilumab, which have been anecdotally used in irAE (Stroud et al. 2019), could have a broader role.

Lately, our group has shown a correlation between the acute onset of an irAE with the rise in CRP levels (Abolhassani et al. 2019). IL-6 induces, e.g. in chronic inflammatory diseases, via the JAK/STAT3 pathway the transcription of the CRP gene and, therefore, acute phase expression of CRP (Nishikawa et al. 2008). This is especially interesting, since Yoshida et al. (2020) demonstrated that high CRP levels and other acute phase proteins suppress adaptive immunity and therefore exhibit an immunosuppressive function that also inhibits tumor response of melanoma patients. This implies that a preliminary combination of ICI with anti-IL-6R antibodies would facilitate a better responsiveness to the administered ICI therapy. Similarly, enhanced IL-8 (CXCL8) levels have shown to worsen the clinical outcome of ICI therapy, in this case PD-L1 antibody atezolizumab (Yuen et al. 2020). It has also been shown that reduced baseline levels for CXCL9, CXCL10, CXCL11 and CXCL19 (Khan et al. 2019) and elevated IL-6 baseline levels can be used as predictors for irAE (Valpione et al. 2018). In a study of 40 different chemokines, it could be shown that 2 and 6 weeks post-treatment CXCL9 and CXCL10 increased in patients with irAE (Khan et al. 2019). This increase of CXCL10 falls in line with our results of a significant increase of IL-6 and CRP, since it is known that IL-6 stimulates the release, among others, of CXCL10 and directs T-cell infiltration in tissue via trans-signaling (McLoughlin et al. 2005).

In conclusion, the inflammatory processes preceding the clinical presentation of irAEs involve an increase of IL-6. Further studies to characterize the cytokine network and to establish reliable biomarkers for irAE are needed.

## Data Availability

The datasets generated during and/or analyzed during the current study are available from the corresponding author on reasonable request.
